# Case of Scorbutus, Combined with Dyspepsia

**Published:** 1800-12

**Authors:** 


### *Case of Scorbutus, combined with Dyspepsia.*

I firft faw the Rev. Mr. H. a clergyman, refident about eight miles from this place, on the 5th of April, 1800. He then complained of great laffitude and debility, of pain and rigidity in the lower extremities, which were almoft entirely covered on the fore part with large vibices of various colours, particularly brown and purple. Towards evening the legs were œdematous. The gums had juft begun to be fore, painful, and to bleed upon being flightly touched. His nights were reftlefs, and the fleep he got not refrefhing. His pulfe was fmall and quick, his bowels loofe, his appetite very bad; he was frequently affected with gaftrodynia, flatulence, and uneafinefs after taking food. Upon enquiry into the previous hiftory of the difeafe, I found that he had laboured under dyfpepfia for feveral years, and that his bowels had been fo much difordered that for two years he had not had a naturally-formed ftool. He had not dared to eat vegetables for near two years paft; his diet had principally confifted of toafted bread, gruels, and flefh meat, but of thefe he had eaten very fparingly, on account of the uneafinefs which he experienced after taking them, as well as from anorexia. He led a fedentary life, and had been under the influence of grief for fome time paft. The whole of this cafe being recorded from memory, fome time after the event, I cannot vouch for the accuracy of every minute particular, but am certain that the general ftatement will be found to be correct. The whole clafs of remedies neceffary for the cure of fcurvy difagreeing with my dyfpeptic patient, I felt fome difficulty in prefcribing for him. Convinced, however, that the only chance for faving his life confifted in conveying remedies into the fyftem, containing oxygen in a ftate eafily to be decompofed, in order to enable the ftomach to retain and digeft fuitable diet, I prefcribed the following medicine:

℞. Decoct.

℞. Decoct. cort. cinchon. ℥vij. Tinct. cort. cinchon. comp. ℥j. Confect. aromat. ʒij vel ʒiij. Tinct. opii gt. LXXX. M. ft. Apozem. partitis vicibus quotidie ſumend.

I ordered him to have a pint of Port wine, and to eat half a dozen oranges daily; to take ſome good broth, mixed with the juice of nettles (the only vegetable then exiſting in that part of the country) and to eat broiled meat once or twice a day, according as the appetite would allow.

In caſe diarrhœa ſhould recur during the uſe of the above remedies, I directed Mr. H. to take a tea ſpoon full of infuſion of logwood, with a few drops of tinct. opii after every looſe ſtool.

I ſaw Mr. H. again on the 10th of April, when I had the ſatisfaction of finding him, in every reſpect, much better. The quantity of tinct. opii, of courſe, occaſioned a good deal of drowſineſs, but it enabled his ſtomach to retain both food and medicines. The ſtrength and appetite had increaſed very much; his ſtools were regular and well formed; his pulſe was ſlower and ſtronger. I could not obſerve much alteration in the appearance of the blotches, but the gums were leſs ſore, and the limbs leſs painful. I ordered his medicines to be continued, diminiſhing the quantity of tinct. opii to ſixty drops daily; he never had occaſion to uſe the logwood. I directed him to take ſuch freſh vegetables as the ſeaſon afforded.

On the 5th of May I found that he had continued gradually and regularly to recover ſince my laſt viſit. His appetite for food, both animal and vegetable, had become very keen, and he had eaſily digeſted it. He had gained much ſtrength and fleſh, and his pulſe was regular. Many of the vibices on the extremities had diſappeared, and ſuch as remained, inſtead of being purple and brown, had become yellow. He now nearly diſcontinued his medicines, ſtill, however, perſevering in the uſe of a generous diet.

The hiſtory of ſcurvy originating from inſufficient vegetable nouriſhment, has been ſo well explained and exemplified by Drs. Milman and Storke, that I ſhould have conſidered the recitation of this caſe totally unneceſſary, had it not been on account of its complication with dyſpepſia. Independent of the cure of the ſcorbutic ſymptoms, the relief that was afforded to the dyſpepſia was greater than could have been calculated upon in the given time. A day or two before I ſaw him, Mr. H. eat part of an orange, which occaſioned much uneaſineſs in the ſtomach and bowels. Yet, a ſhort time after I ſaw him, when under the influence of opium, the primæ viæ were able to digeſt daily, without annoyance, ſix oranges, beſides broth, with nettle juice, animal food, and wine. In caſe the opium had not produced the defired effect, the inhalation of oxygen gas would have been the only probable mean of faving life; but, from the fituation in which my patient was placed, the adminiftration of it would have been attended with much trouble and inconvenience.

Two other inftances have occurred to me, within the laft twelve months, of fcurvy produced by a meagre and infufficient diet, which readily gave way to the ufual remedies.

